# Effects of change in dysfunctional beliefs in avatar-based cognitive therapy for depressive symptoms: a randomized parallel trial

**DOI:** 10.1038/s41598-025-96228-8

**Published:** 2025-06-04

**Authors:** Nicolina Laura Peperkorn, Julia Ohse, Janosch Fox, Merle Kuhlencord, Christin Janine Grevenhaus, Joshua Krutzki, Michael Witthöft, Youssef Shiban

**Affiliations:** 1 Clinical Psychology Department, PFH Private University of Applied Sciences, Göttingen, Germany; 2 Department of Clinical Psychology, Psychotherapy and Experimental Psychopathology, Johannes Gutenberg University Mainz, Mainz, Germany

**Keywords:** Avatar, Digital intervention, Dysfunctional beliefs, Depressive symptoms, Confrontation, Psychology, Health care, Human behaviour

## Abstract

This study evaluated the effect of an avatar-based intervention on depressive symptoms and self-esteem. Participants (*N* = 151) with subclinical depressive symptoms were instructed to challenge an avatar over three sessions. While participants within the intervention group challenged their personal dysfunctional beliefs, participants in the control group challenged nonsense statements. Allocation to treatment groups was randomized. Data collection took place pre-intervention and post-intervention. Statistical analysis revealed a significant decrease in depressive symptoms, which was more pronounced for the intervention group (*p* < .01), as well as a significant group × time interaction for self-esteem (*p* < .05). The effect on depression symptom strength was large in the experimental group (*d* = − 1.19) and medium (*d* = − 0.72) in the control group, while the effect on self-esteem was moderate (*d* = 0.54) in the intervention and small (*d* = 0.29) in the control group. Our findings on symptom reduction align with prior research, while positive effects on self-esteem are a novelty. These results demonstrate the intervention’s potential for reducing the symptoms of mental illness.

## Introduction

Depressive disorders are among the most prevalent mental health conditions, affecting over 10% of the global population^1,2^. When depression is assessed with self-report measures, the prevalence rate rises to 17.3%, hinting at a higher depressive burden within the community that does not necessarily meet diagnostic criteria for depressive disorder^1^.

Cognitions are of decisive importance in the onset and persistence of depression, forming the foundation of Cognitive Therapy (CT) and various digital mental health interventions^2–6^ Cognitions shape the perceptions of oneself, others, and the world while mostly focusing on themes of self-worth and autonomy^7^. For example, the cognition of oneself as a worthless or inadequate individual biases the interpretation of events. which leads to pervasive negativity and the perception of failure^8,9^. These negative self-perceptions manifest in low self-esteem, driven by distorted cognitive patterns, that result in negative conclusions about one’s own value^10^. Multiple different models propose a connection between self-esteem and depressive symptoms (see Orth & Robins^11,12^). Evidence^13^ from longitudinal studies points towards a stronger effect of self-esteem on depression than vice versa^14^, which is in line with Fennel’s cognitive conceptualization of low-self-esteem^15^. Self-esteem has been found to mediate the relationship between dysfunctional thoughts and depressive symptoms^13^. This warrants a closer investigation of self-esteem as a potential outcome of CTs, like the avatar therapy, in which dysfunctional cognitions and their alteration present the primary objective^14^.

Recent research has demonstrated that the modification of such dysfunctional cognitions through avatar-based digital interventions can significantly reduce symptom severity in patients experiencing depression^16^: In their study, Kocur et al.^16^ tested a computer-assisted avatar-based treatment for dysfunctional beliefs (CAT-DB) on a sample of inpatients experiencing severe depression. During this treatment, a virtual avatar confronted the patients with their dysfunctional beliefs. The avatar would state a patient’s dysfunctional beliefs, while the patient was tasked with challenging these beliefs with congruent, functional alternative thoughts. Compared to the treatment as usual (TAU) group, the experimental group showed a significantly greater reduction in dysfunctional cognitions and overall symptom severity at post-intervention. Notably, these improvements persisted and remained stable during a follow-up assessment two weeks later. These results show great promise for the avatar intervention as a potential treatment enhancement for individuals with severe depression in a controlled in-patient setting. However, with high prevalence rates of depressive symptoms in the general population, assessing the potential of this intervention in individuals with milder symptoms, who are currently not in treatment, might be of interest. Since the avatar treatment can be delivered digitally and automated, it offers a high potential for dissemination, which might offer a small contribution to closing the treatment gap. Particularly so as early intervention in subclinical populations has been shown to prevent the progression to clinical depression and reducing the overall burden on healthcare systems^17,18^.

The assumption, that the avatar-intervention might show positive effects in this population is based on the results of a meta-analysis by Cuijpers et al.^18^, which demonstrates that interventions for individuals experiencing strong depression symptoms are effective in lowering less severe depressive symptoms as well.

In line with this, Fey et al.^19^ observed positive effects when using CAT-DB in participants with subclinical eating disorder symptoms. The CAT-DB group exhibited significantly greater reductions in eating disorder specific symptoms compared to the control group at the follow-up assessment two weeks after the intervention. While these results were obtained for dysfunctional cognitions in another mental disorder than depression, this underlines the potential of the avatar intervention for altering cognitions in a subclinical mental disorder context. For subclinical depression, however, a thorough examination is still pending^17,18,20^.

The overall effectiveness and general user acceptance of digital interventions with an avatar-based approach have been well documented^5,6,21–26^. Particularly digital interventions grounded in Aaron T. Beck’s Cognitive Theory have shown favourable results^5,6^, which lend optimism towards the potential of a digital, automated intervention as a vehicle for the dissemination of the avatar-based intervention.

The current study employs a digital, avatar-based intervention with undiagnosed individuals experiencing depressive symptoms, who are currently not undergoing therapeutic treatment. Building upon the findings of Kocur et al.^16^ and Fey et al.^19^, our goal is to discern whether the intervention can decrease depressive symptoms while increasing self-esteem. For this, we hypothesize the following:

1. The intervention group will experience a significantly greater decrease in depressive symptoms throughout the intervention, compared to the control group.
2. The intervention group will show a significantly greater increase in self-esteem throughout the intervention, compared to the control group.

## Methods

### Study design outcomes

The study was carried out as an experimental design with two parallel treatment groups and random 1:1 allocation of study participants. For the outcome variables “depressive symptom strength” and “self-esteem”, we utilized the German versions of the Beck Depression Inventory-II (BDI-II^27^) and the Rosenberg Self Esteem Scale (RSES^28^).

#### Materials

The ICD-10 Symptom Rating (ISR) is a self-report measure and features 6 subscales (depression, anxiety, obsessive/compulsive, somatoform, eating disorders, and the supplementary scale). It comprises 29 items, each rated on a five-point Likert scale (0 = does not apply, 1 = hardly applies, 2 = fairly applies, 3 = strongly applies, 4 = extremely applicable)^29^. The depression subscale consists of four items. It has been thoroughly tested for reliability and validity with a sample of 3,755 participants^30^. Satisfactory retest-reliability (*r* = 0.78) of the depression subscale in a subclinical sample, together with its sensitivity to change brought on by psychosomatic treatment^31^ were factors in our decision to use the ISR depression subscale for the screening.

The BDI-II is a self-assessment tool for measuring depressive symptom strength. It consists of 21 items, taking approximately 5–10 min to complete. It is suitable for individuals aged 13 and above^32^. Each item pertains to a different depression symptom and consists of four responses describing the symptom manifestation in four gradations, ranging from 0 to 3, each. The four gradations depict different levels of symptom severity as expressed by frequency, duration and/or intensity of the symptom as experienced in the past 14 days. The questionnaire is answered by marking the answer the participant deems as most descriptive of their experience^32^. The BDI-II is evaluated by building a mean score which can range from 0 to 63. The German national care guideline for unipolar depression^33^ recommends cut-off scores for different categories of depression severity. Retest-Reliability was found to be very good (0.78); internal consistency was 0.90 within a non-clinical sample^34^. Due to its sensitivity to change, the BDI-II has become a gold standard to measure symptom severity in depression in clinical settings, as well as in research.

The RSES (German version by von Collani & Herzberg^28^) is a self-report measure to assess self-esteem, consisting of 10 items rated on a four-point Likert-scale. Each item represents a statement that relates to one’s self-worth, such as the feeling of being a person of worth. Participants rate their agreement with the item’s statement on a 4-point Likert-scale which is scored between 0 and 3. As 5 of the items include negative self-worth statements, they are reverse-coded for the calculation of the sum score, which ranges from 0–30. An RSES-score below 15 indicates low self-esteem. Internal consistency (α > 0.80^35^ and reliability (0.82–0.84^35^) can be considered as high.

### Intervention

**Fig. 1:**
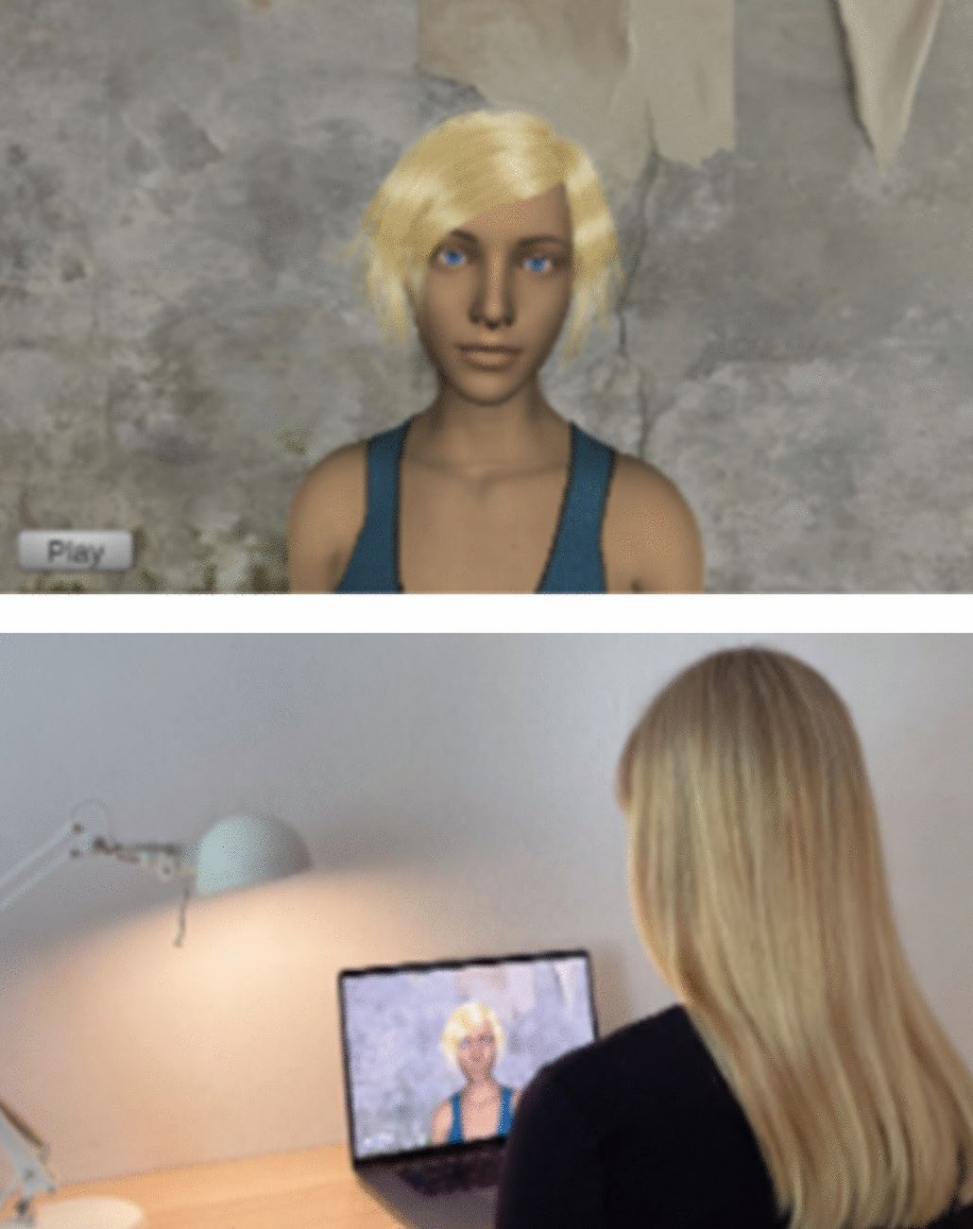
Interface and experimental setup.

The intervention was delivered by an avatar. The interface featured an outwardly female-looking avatar placed in the center of the screen, displayed from a head-and-shoulder perspective. In the left-hand corner of the interface, there is a play button that allows participants to start the intervention independently. The experimental setup consists of a laptop that provides access to the intervention through a web browser The interface of the avatar and an example of an experimental setup can be seen in Fig. 1.

The digital intervention was created using the Unity3D engine, with the avatar realized using Daz 3D’s Genesis 8 model. Talking animations were realized with the SALSA LipSync v2 Suite. The avatar is portrayed from head to bust and depicted as female-presenting, caucasian, around 20–40 years old and dressed in casual clothing. Dysfunctional beliefs and nonsense statements were uttered in a neutral tone, with a neutral facial expression while pertaining eye contact with the participants.

Similar to the studies by Kocur et al.^16^ and Fey et al.^19^, participants within the intervention group were tasked with interacting with the avatar presenting them with their own dysfunctional beliefs and positive alternatives. Said beliefs were collected from the participants after an initial psychoeducational session on dysfunctional beliefs. In contrast to this, the control group was presented with nonsense statements bearing no emotional valence (e.g. “Hamburg is the capital city of Germany”). Both groups were tasked with contradicting the statements made by the avatar. For the control group, the contradiction included alternative, functional beliefs (e.g. “That is not true, my best friend likes me.”) or true statements (e.g. “That is not true, Berlin is the capital city of Germany.”).

Our intervention differed from that of Kocur et al.^16^ in that it was delivered in an automated manner, via web application, requiring no experimenter supervision. Participants were free to start the intervention by themselves at any given moment within the testing period, by accessing the application in a web browser. The structure of our intervention was streamlined, consisting of three sets of three confrontations, with each session lasting 10–15 min.

### Ethics

This study has been preregistered in the German Clinical Trials Register (DRKS) on August 13th, 2024, under the DRKS-ID: DRKS00034832. Prior to participation, participants received written information on the purpose of the experiment, on the intervention and questionnaire process, as well as the collection, storage, and protection of their data. Informed consent for the participation in the experiment as well as for the collection of data and data processing was obtained in line with the Declaration of Helsinki. The Ethics Committee of the PFH Private University of Applied Sciences Göttingen was informed of the process and approved the research protocol under YS_23_270622 prior to the start of data collection. Adherence to GDPR regulations was ensured by using the online survey platform LimeSurvey for data collection. The questionnaires were designed to collect only the essential data, in line with the principle of data minimization. Participants were informed of their rights to access, revise, delete and restrict the use of their personal data, as outlined in the consent form. To maintain privacy, data were pseudonymized at collection, with identifying information stored separately from study data. All data was handled fairly, ethically and transparently, with appropriate security measures in place.

### Process

Recruitment was conducted via email lists, social media, and university forums, with informed consent obtained from all participants. The study comprised several stages: a dual screening-process, pre-treatment assessment, three intervention sessions with the avatar, and a 14-day post-assessment.

The first stage of the screening-process included a demographic questionnaire and the ISR^29^, both to be filled out online, with time and location freely determined by the participant. The demographic questionnaire covered visual and auditory disabilities, German language proficiency, history of psychotic or manic symptoms as well as current psychotherapy. Respondents under the age of 18 were excluded. Furthermore, respondents whose experience of the intervention would be impacted by visual/auditory disability or lack of German language proficiency were excluded. Respondents with past episodes of psychosis or mania, as well as respondents currently undergoing psychotherapeutic treatment were excluded for ethical reasons, to not interfere with their ongoing therapy, to ensure the exclusion of confounding variables and accurate inclusion of the sample population. The ISR was used to identify comorbidities, by accessing symptomatology across six subscales, including depression, anxiety, and eating disorders. A score below 0.75 on the depression subscale was used as an exclusion criterion, as participants not experiencing any form of depressive burden would not benefit from the intervention. Additionally, the BDI-II, as a disorder-specific screening-tool, was employed. A cut-off score of greater than 13 was defined, indicating mild depressive symptoms.

Participants determined eligible for the experiment were randomly assigned to either the intervention or control group. Randomization was carried out by assigning unique numbers to participants and distributing these numbers to either of the two intervention groups in a 1:1 ratio using an online tool. Following this procedure, eligible participants received a standardized email with a link either leading to the experimental intervention or leading to the control intervention. Participants did not receive any information on (a) the existence of two different intervention groups or (b) which group of the experiment they were in. The emails were sent by different experimenters working within the project. While experimenters were not blinded towards which group their participants were in, they had no direct interaction with the participants as the experiment was carried out online and instructions were given in standardized texts.

The pre-measurement contained a psychoeducational session on dysfunctional beliefs for both groups. The aim of this was to enable participants to identify their individual dysfunctional thoughts. Psychoeducation was delivered via a digital brochure. Following this, participants were instructed to write down three of their individual dysfunctional beliefs. During the pre-measurement, the BDI-II and RSES were administered, followed by the first avatar intervention session. The second and third intervention sessions with the avatar took place on the following two days. Fourteen days after the last intervention session, the post-measurement was conducted, during which the BDI-II and RSES were administered once more.

All questionnaires and interventions were delivered online, so that participants could choose the time and place of their participation, limited only by the times at which new questionnaires and interventions were provided. Data was collected at PFH Göttingen, Germany.

The study procedure can be seen in detail in the Supplementary Figure S1.

### Statistical analysis

Data analyses were performed using R 4.0.4. The directional hypotheses were tested using a two-factor repeated measures analysis of variance (2 × 2 ANOVA), with the between-subjects factor treatment group (intervention group [*n* = 80] vs. control group [*n* = 71]) and the within-subjects factor time (pre- and post-intervention). The dependent variable for the first hypothesis was depressive symptom strength as measured by the BDI-II-score, the dependent variable for the second hypothesis was self-esteem as measured by the RSES-score. We decided to perform an intention-to-treat analysis (ITT) using the Last Observation Carried Forward (LOCF) method, to evaluate the robustness of our findings. A systematic outlier detection and exclusion based on the IQR method was performed and led to the exclusion of *n* = 12 participants.

## Results

### Sample

Based on previous work by Kocur et al.^16^ and Fey et al.^19^, we calculated the required sample size using G*Power^36^ for a smaller effect size of Cohen’s f = 0.15 with a power (1 –β) of 0.95 and an α-error-probability of 0.05. Power analysis revealed that *N* = 148 participants were necessary to achieve these criteria.

A sample of *n* = 1395 participants was screened. Data from this sample was evaluated for pre-screening exclusion criteria, resulting in a total of *n* = 662 subjects being excluded from the study. Between pre-measurement and final data analysis, *n* = 482 participants were excluded, due to (a) an ISR score ≥ 0.75 and a BDI-II score below the cut-off value of 13 (*n* = 803), (b) not finishing the experiment (drop-out) (*n* = 163, 54.6% of all eligible subjects) or (c) outlier values according to IQR (*n* = 12). The flow chart of the study’s experimental procedure, including the dropout, can be seen in Supplementary Figure S2.

This results in a total sample of *N* = 151 which was analyzed for this study. Further details regarding this procedure are- depicted in Supplementary Figure S2 in the appendix.

Participants were randomly assigned to either the intervention group (*n* = 80, 56 women, 23 men, 1 diverse, *M*_age_ = 31.3, *SD*_age_ = 10.5) or the control group (*n* = 71, 50 women, 21 men, 0 diverse, *M*_age_ = 29.2, *SD*_age_ = 10.8). Baseline comparisons confirmed the absence of significant differences in gender distribution between the intervention- and control-group. Regarding the highest educational qualification, having a completed university degree was dominant with *n* = 40 subjects. Sociodemographic characteristics can be found in Table 1.

**Table 1:** Sociodemographic characteristics of the sample.

	EG		CG		Total sample	
	*n*	%	*n*	%	*n*	%
Gender						
Female	56	70	50	70.42	106	70.19
Male	23	28.75	21	29.58	44	29.14
Diverse	1	1.25	0	0	1	0.66
Education						
University degree	40	50	23	32.39	63	41.72
University qualification	21	26.25	28	39.44	49	32.45
Completed training	17	21.25	17	23.94	34	22.51
Other	2	1.25	3	4.22	5	3.31

EG = intervention-group. CG = control-group. *N* = 151.

### H1 Greater decrease in depressive symptoms within the intervention group

**Fig. 2:**
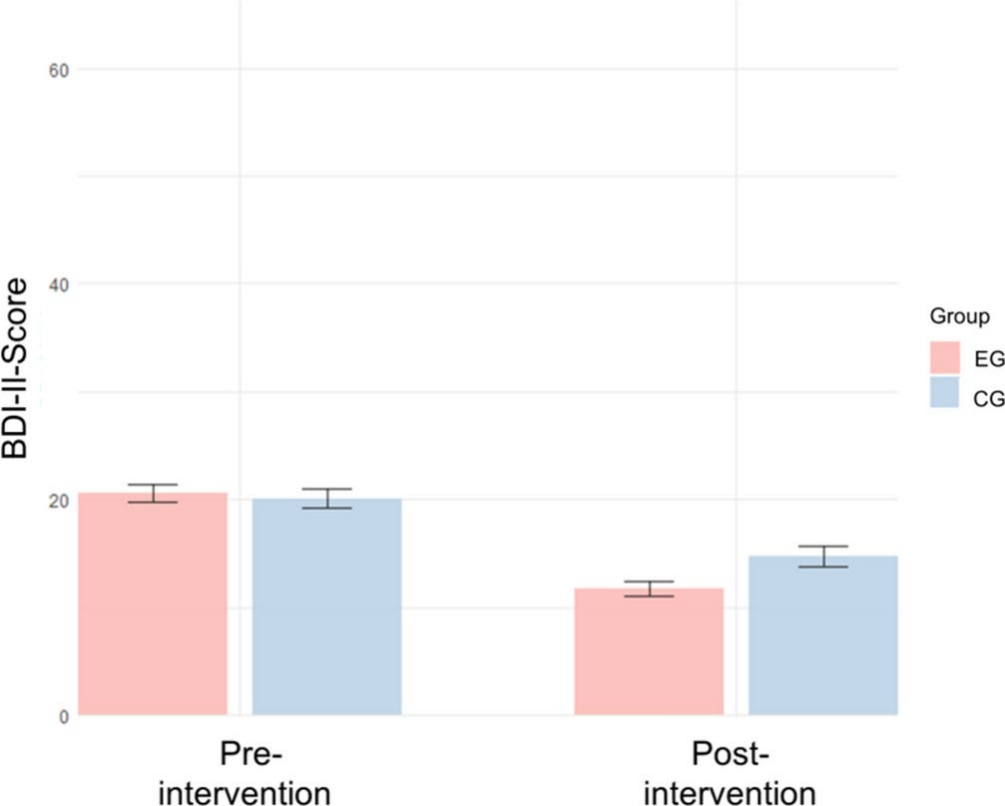
BDI-II scores for the intervention- and control-group, pre- and post-intervention. *Note* Mean scores of the Beck-Depression-Inventory II (BDI-II) for the intervention-group (EG; *n* = 80) and the control-group (CG; *n* = 71). Error bars show standard errors.

Consistent with hypothesis 1, Fig. 2 shows the decrease in depressive symptoms, as measured with the BDI-II, for both the intervention group (EG; *n* = 80) and the control group (CG; *n* = 71) pre- and post-intervention.

#### Descriptive data

As shown in Fig. 2 the pre-intervention BDI-II-score was *M* = 20.6 (*SD* = 7.10) for the intervention group (n = 80) and *M* = 20.1 (*SD* = 7.19) for the control group (n = 71). This indicates a moderate severity of depression, according to the cut-off criteria promoted by the German National Care Guideline for unipolar depression^33^. To confirm the results were not biased by pre-existing differences, we compared the BDI-II scores between the intervention and control groups at baseline. The statistical analysis revealed no significant differences (Welch two-sample t-test: *p* = 0.841). This indicates that both groups had a comparable baseline severity of depressive symptoms before the intervention. At post-intervention, the mean BDI-II-score in the intervention group was *M* = 11.8 (*SD* = 6.77), while the mean score in the control group was *M* = 14.8 (*SD* = 8.02), as depicted in Table 2, showing a decrease of 8.8 points within the intervention group and a decrease of 5.3 points within the control group. The calculated effect sizes show a large effect for the reduction of depression symptoms in the intervention group (*d* = − 1.19) and a moderate effect size for the reduction of depression symptoms in the control group (*d* = − 0.72).

**Table 2:** Descriptive metrics for the BDI-II- and RSES-Scores.

Variables	EG		CG	
	M	SD	M	SD
BDI-II				
Pre intervention	20.6	7.10	20.1	7.19
Post intervention	11.8	6.77	14.8	8.02
RSES				
Pre intervention	16.4	5.56	15.3	6.23
Post intervention	19.2	4.97	16.8	5.50

EG = intervention group. CG = control group. *N* = 151. Baseline comparisons showed no significant differences between groups in BDI-II (*p* = 0.841) and RSES (*p* = 0.258), confirming group equivalence prior to intervention.

#### Inference statistics

A 2 × 2 repeated measures ANOVA, with treatment group as the between-subjects factor, time as the within-subjects factor and depressive symptom strength (BDI-II), revealed a statistically significant interaction effect, *F*(1, 149) = 9.518, *p* < 0.01, *η2* = 0.014. Furthermore, a significant main effect for the within-subjects factor time was revealed, *F*(1, 149) = 159.958, *p* < 0.001, *η2* = 0.194. However, no effect was found for the factor group, *F*(1, 149) = 1.453, *p* > 0.05, *η2* = 0.008. The interaction effect between group and time point remained significant in the ITT analysis with the LOCF method (*F*(1, 155) = 5.820, *p* = 0.017), indicating that the inclusion of participants with missing data did not alter the overall results for the BDI-II. A visualization of these results can be seen in Fig. 2.

### H2 Greater increase in self-esteem within the intervention group

**Fig. 3:**
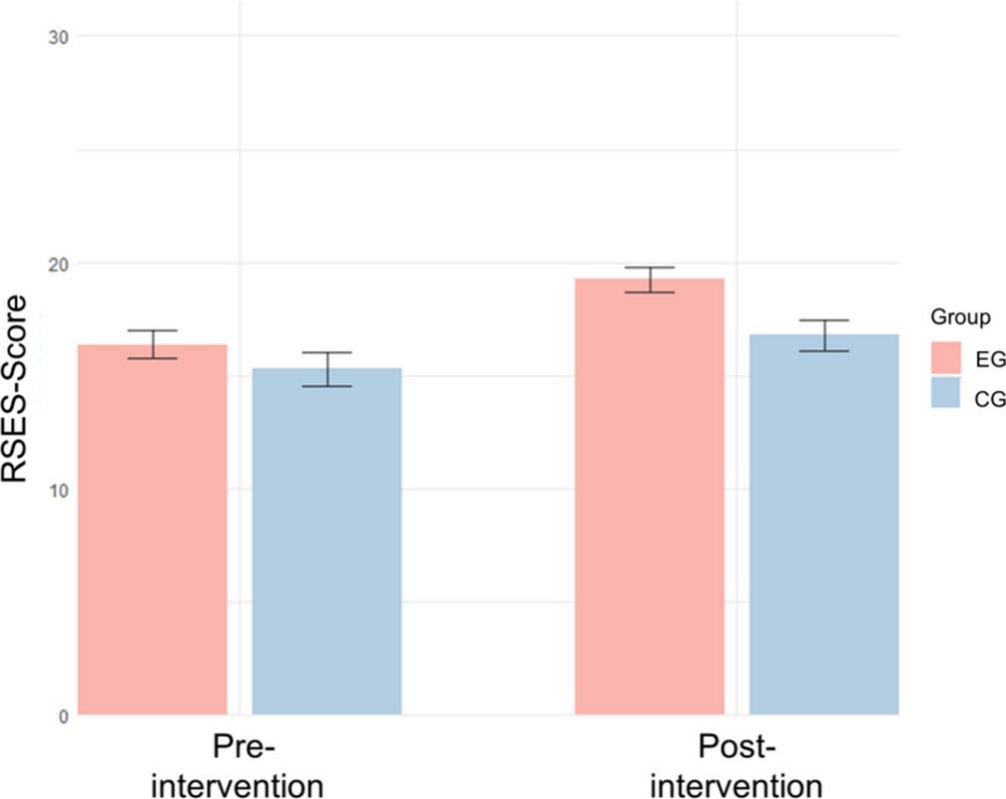
RSES-scores for the intervention- and control-group pre- and post-intervention. *Note* Mean scores of the Rosenberg Self Esteem Scale (RSES) for the intervention-group (EG; *n* = 80) and the control-group (CG; *n* = 71). Error bars show standard errors.

In line with hypothesis 2, Fig. 3 illustrates the increase in self-esteem, as measured by the Rosenberg Self-Esteem Scale (RSES), for both the intervention group (EG; n = 80) and the control group (CG; n = 71) pre- and post-intervention.

#### Descriptive data

Section "Descriptive data" presents the descriptive data to provide context for the subsequent inferential statistical analysis. Pre-intervention, participants in the intervention group had a mean RSES-Score of *M* = 16.4 (*SD* = 5.56). In the control group, participants reported an RSES-Score of *M* = 15.3 (*SD* = 6.23). This can be seen in Fig. 3. The comparison of baseline RSES scores between the intervention- and control-groups showed no significant differences (two-sample Welch t-test: *p* = 0.258), confirming that the level of self-esteem before the intervention was comparable between the groups. Post-intervention (14 days past intervention), the RSES-score in the intervention group had risen to *M* = 19.2 (*SD* = 4.97), while the RSES-score in the control group had increased to *M* = 16.8 (*SD* = 5.50), as depicted in Table 2. The calculation of the effect sizes resulted in a moderate effect (*d* = 0.54) in the intervention group and a small effect (*d* = 0.29) in the control group.

#### Inference statistics

Regarding the RSES, a 2 × 2 repeated measures ANOVA, with treatment group being the between-subjects factor and time being the within-subjects factor, revealed a statistically significant interaction effect, *F*(1, 149) = 5.216, *p* < 0.05, *η2* = 0.004. Furthermore, a significant main effect for the within-subjects factor time was revealed, *F*(1, 149) = 52.574, *p* < 0.001, *η2* = 0.038. In addition, a significant main effect for the factor group was revealed, *F*(1, 149) = 4.205, *p* < 0.05, *η2* = 0.004. While the main effects remained significant in ITT analysis using the LOCF method, the interaction effect between group and time was no longer significant (*F*(1, 155) = 2.764, *p* = 0.098). A visualization of these effects can be seen in Fig. 3.

## Discussion

The aim of this study was to evaluate the effect of an avatar-based intervention on depressive symptoms and self-esteem in an undiagnosed population sample experiencing depressive symptoms, that is currently not in psychotherapeutic treatment.

Our findings provide initial evidence for the effectiveness of the avatar-based intervention in reducing depressive symptoms and increasing self-esteem. The significantly greater reduction in depressive symptoms in the intervention group supports our hypothesis that targeting and contradicting individual dysfunctional beliefs is more effective than contradicting nonsense-statements. The large effect size (*d* = − 1.19) for the intervention group compared to the moderate effect size (*d* = − 0.72) for the control group further underlines the effectiveness of the cognitive intervention delivered by the avatar. The significantly greater increase in self-esteem in the intervention group indicates that addressing individual dysfunctional beliefs through the avatar-based intervention not only reduces depressive symptoms but also contributes to an increase in self-esteem. The moderate effect size (*d* = 0.54) within the intervention group compared to the small effect size (*d* = 0.29) in the control group provides additional support for this finding.

To assess the robustness of these findings, we conducted an ITT-analysis using the LOCF-method. The results suggest that the inclusion of participants with missing data did not alter the overall results for depressive symptoms. However, while the main effects for self-esteem remained significant in the ITT analysis, the interaction effect between group and time was no longer significant. These findings indicate the increase in self-esteem may require further investigation.

Our findings are in line with those of Kocur et al.^16^ while following their suggestion of evaluating dysfunctional cognitions and adding self-esteem as an outcome variable. Notably, our research featured a sample without a diagnosis and currently not undergoing psychotherapeutic treatment, proving an effectiveness of the avatar-based treatment for less pronounced depressive burden than to be found in a sample of inpatients, as evaluated by Kocur et al.^16^. Therefore, it is of no surprise that the effect sizes in our study are lower than those obtained by Kocur et al.^16^. This is in line with the results from a metaanalysis^18^ demonstrating that interventions designed for clinical depression are also effective in samples with subclinical depression, while yielding lower effect sizes. Consequently, our results align with empirical findings proving the effectiveness of avatar-based interventions in different mental illnesses^16,19,37–40^. In contrast to Kocur et al.^16^, who did 3 × 10 confrontations per setting, we found evidence for the effectiveness of the avatar-based intervention with just 3 × 3 confrontations per setting. This is in line with the findings of Schleider et al^41^, who found their avatar-based intervention to be effective in a single session. All in all, session duration should be explored in future research, as these results point towards the potential of extremely short interventions.

We also address the potential cost-effectiveness and accessibility of our intervention. We acknowledge that we cannot provide concrete evidence of cost-effectiveness at this stage of our study. However, studies from other areas, such as eating disorders^19^ or depression^16^, suggest potential cost savings due to similar interventions. In addition, a study by Hedman et al.^42^ demonstrates the effectiveness of internet-based cognitive behavioral therapy (iCBT) compared to traditional cognitive behavioral therapy (CBGT) in the treatment of social anxiety disorder. Their findings suggest that iCBT offers similar levels of improvement in reducing social anxiety, while being more cost-effective, flexible and allowing participants to work at their own pace. We recommend that this aspect be investigated further in future research.

Future research should also evaluate the effects of participants’ acceptance of virtual interventions, as there is evidence for this being a considerable influence on the effectiveness of treatment^43^. Another factor requiring more research is avatar appearance. Within this study, a female presenting avatar was used. However, with evidence pointing towards avatar modification increasing the willingness to show effort in virtual interventions^22^, there might be potential for increasing therapy effects through such a component. Dechant et al.^44^ show that the ability to customize the avatar significantly increases user engagement and social presence. These aspects are essential factors for effective treatment in digital therapy. Furthermore, Reichenberger et al.^45^ emphasize the importance of agent characteristics in triggering emotional responses and show that women tend to experience higher anxiety levels than men when interacting with male avatars during fear conditioning in virtual reality. Furthermore, Ashrafi et al.^46^ found that avatar characteristics have a significant impact on intervention success and demonstrated differences in emotional well-being and motivation between self-similar and dissimilar virtual agents. These results highlight that identification, especially when enhanced by customization, can increase motivation for an interaction with the avatar. By integrating these findings, future studies can better understand how avatar characteristics influence therapy outcomes and improve the overall effectiveness of digital interventions.

## Limitations

A counter-intuitive finding from our study is that participants in the control group also appeared to profit from their intervention, which did not include personal dysfunctional beliefs. Reasons for that might be (a) that the act of contradicting the avatar itself has potential for empowering self-esteem and lowering depression values (e.g. through self-efficacy), (b) that the psychoeducation on dysfunctional cognitions serves as an intervention, (c) placebo-effect or (d) demand characteristics. It is known from clinical practice that patients with low self-esteem have difficulties with self-assertiveness, especially if they must refuse an offer or a task or if they have to correct others. Simply practicing to “say no” is a concept exercised by behavioral therapists. Therefore, contradicting the avatar might have already influenced self-esteem while the self-efficacy might have impacted depression scores. Another limitation of our study lies in the element of psychoeducation. While previous research has shown that psychoeducation can significantly influence mental health outcomes^47^, this component was not systematically examined in our study. Nevertheless, our randomized controlled trial (RCT) design ensured that psychoeducation was equally distributed across both groups, minimizing potential confounding effects. Despite its presence, we still observed significant effects of our intervention, suggesting that our findings extend beyond the general benefits of psychoeducation. To further differentiate these effects, future studies could systematically manipulate psychoeducation, either by examining the intervention without it or by incorporating it as a separate factor in the study design. However, the effect could also—at least partly—be attributed to the placebo effect or demand characteristics, which made participants score lighter depression values after the intervention. Despite this, it should be noted that the interaction effect still yielded significant results, which speaks for the effectiveness of the cognitive intervention going beyond the element of contradiction. Apart from this, we found no unintended effects. We constantly ensured the safety of the participants during the intervention by providing them with contact information of the project management team. In addition, all participants were provided with an anonymous booklet of external support services, which allowed them to seek help without being identified by the project management team. Although no negative experiences were reported, we would like to point out that a systematic assessment of potential negative effects, e.g. by conducting structured participant feedback, would allow a deeper understanding of potential negative effects in future research.

There was a substantial dropout within this study (*n* = 163, 54.6% of all eligible subjects), for which a systematic loss must be taken into consideration^48^. While the dropout is substantial, the web-based format of the intervention might have prevented even higher drop-out rates, as participants had maximum flexibility regarding when and where to undergo the intervention. At the same time, this might have led to a low degree of perceived social obligation within the participant sample. We suppose that dropout can be influenced by various factors related to the self-administered nature of the intervention. These include participants’ lack of motivation, technical difficulties during the intervention and challenges related to the confrontation with the avatar. As part of future research, it would be beneficial to systematically ask participants about their reasons for dropping out, as this information could provide valuable insights for the developers of future digital and self-administered interventions. Still, with the remaining *N* = 151 subjects, we were able to achieve a high statistical power, which allowed us to test more hypotheses than comparable studies with a smaller sample size (e.g. Kocur et al.^16^, *N* = 34; Fey et al.^19^, *N* = 48).

Another limitation arising from the web-based format is that technical difficulties experienced by the participants could not be directly addressed due to the absence of an experimenter. While subjects could always contact the project leader via email in case of issues or questions, the process of solving these requests was time-delayed. And while potential biases arising from the presence of an investigator were ruled out, the intervention setting was not controlled for and might have posed an influence in some cases.

A technical limitation of this study was restricted browser compatibility, meaning the avatar tool did not function equally well across all web browsers. For future research, as well as for potential practical implementation, it is suggested to implement the avatar in a mobile application, allowing maximum flexibility for the user. The baseline-effectiveness for CBT app-interventions tackling automatic and negative beliefs has already been shown^49^, which lends careful optimism to the potential of a mobile application as an addition to psychotherapy, potentially adding to its treatment effect^50^. Furthermore, there is potential for a mobile application featuring the agent intervention to be used by individuals who experience subclinical depression or are facing barriers to treatment^51^, such as long waiting times^52^.

## Conclusion

This study demonstrates the effectiveness of an avatar-based intervention for lowering symptom severity in participants with subclinical depression, corroborating previous findings for depression^16^, eating disorders^19^, and psychosis^53^. Furthermore, the avatar-based intervention resulted in a significant increase in self-esteem, which is a novel direction for this field of research. Limitations of this study pertain to high dropout rates and technical issues (e.g., browser compatibility issues), which could not be solved directly due to the self-administered nature of the intervention. Future research should explore intervention effectiveness in different application contexts and settings (e.g., clinical, preventive, or as a substitute for regular psychotherapy) as well as varying session duration and frequency. Intervention effectiveness for different mental illnesses and burden levels (clinical/non-clinical) should be topic to further exploration, as the avatar-based intervention offers great potential for dissemination and might offer a contribution to closing the treatment gap for various conditions.

## Supplementary Information

Supplementary Information.

## Data Availability

All (anonymised) data, including the research protocol (in German), will be made available on request per email to the first author.
