# Supplementary material for: Effects of change in dysfunctional beliefs in avatar-based cognitive therapy for depressive symptoms: a randomized parallel trial
Source: Sci Rep. 2025 Jun 4;15:19668. doi: 10.1038/s41598-025-96228-8 (PMC12137557; doi:10.1038/s41598-025-96228-8)
Supplement: Supplementary file 1 — Supplementary Information. [file 41598_2025_96228_MOESM1_ESM.pdf]

Effects of Change in Dysfunctional Beliefs in Avatar-Based Cognitive Therapy for Depressive Symptoms: A Randomized Parallel Trial

Authors

Nicolina Laura Peperkorn[1a] , Julia Ohse[1b], Janosch Fox[1c], Merle Kuhlencord[1d], Christin Janine Grevenhaus[1e], Joshua Krutzki[1f], Michael Witthöft*[2g] Youssef Shiban[1h]

* corresponding author

[1] Clinical Psychology Department, PFH Private University of Applied Sciences, Göttingen, Germany

[2] Department of Clinical Psychology, Psychotherapy and Experimental Psychopathology, Johannes Gutenberg University Mainz, Germany

[a] 0009-0008-9481-9354, peperkorn@pfh.de

[b] 0009-0005-3344-4753, ohse@pfh.de

[c] 0009-0003-2450-5003, fox@pfh.de

[d] kuhlencord@pfh.de

[e] 0009-0009-3747-2603, c.grevenhaus@pfh.de

[f] 0009-0008-1242-9070, joshua.krutzki1@pfh.de

[g] 0000-0002-4928-4222, witthoeft@uni-mainz.de

[h] 0000-0002-6281-0901, shiban@pfh.de

Figure Legends

Figure 1. Interface and experimental setup. *The upper section shows how the avatar appeared on the participant's screen. The lower section shows an example of how the interaction could look like.*

Figure 2. BDI-II scores for the intervention- and control-group pre- and post-intervention. *Mean scores of the Beck-Depression-Inventory II (BDI-II) for the intervention-group (EG; n = 80) and the control-group (CG; n = 71). Error bars show standard errors.*

Figure 3. RSES- scores for the intervention- and control-group pre- and post-intervention. *Mean scores of the Rosenberg Self Esteem Scale (RSES) for the intervention-group (EG; n = 80) and the control-group (CG; n = 71). Error bars show standard errors.*

Table 1. Sociodemographic characteristics of the sample. *EG = intervention-group. CG = control-group. N = 151.*

Table 2. Descriptive metrics for the BDI-II- and RSES-Scores. *EG = intervention group. CG = control group. N = 151.*

Supplementary Information

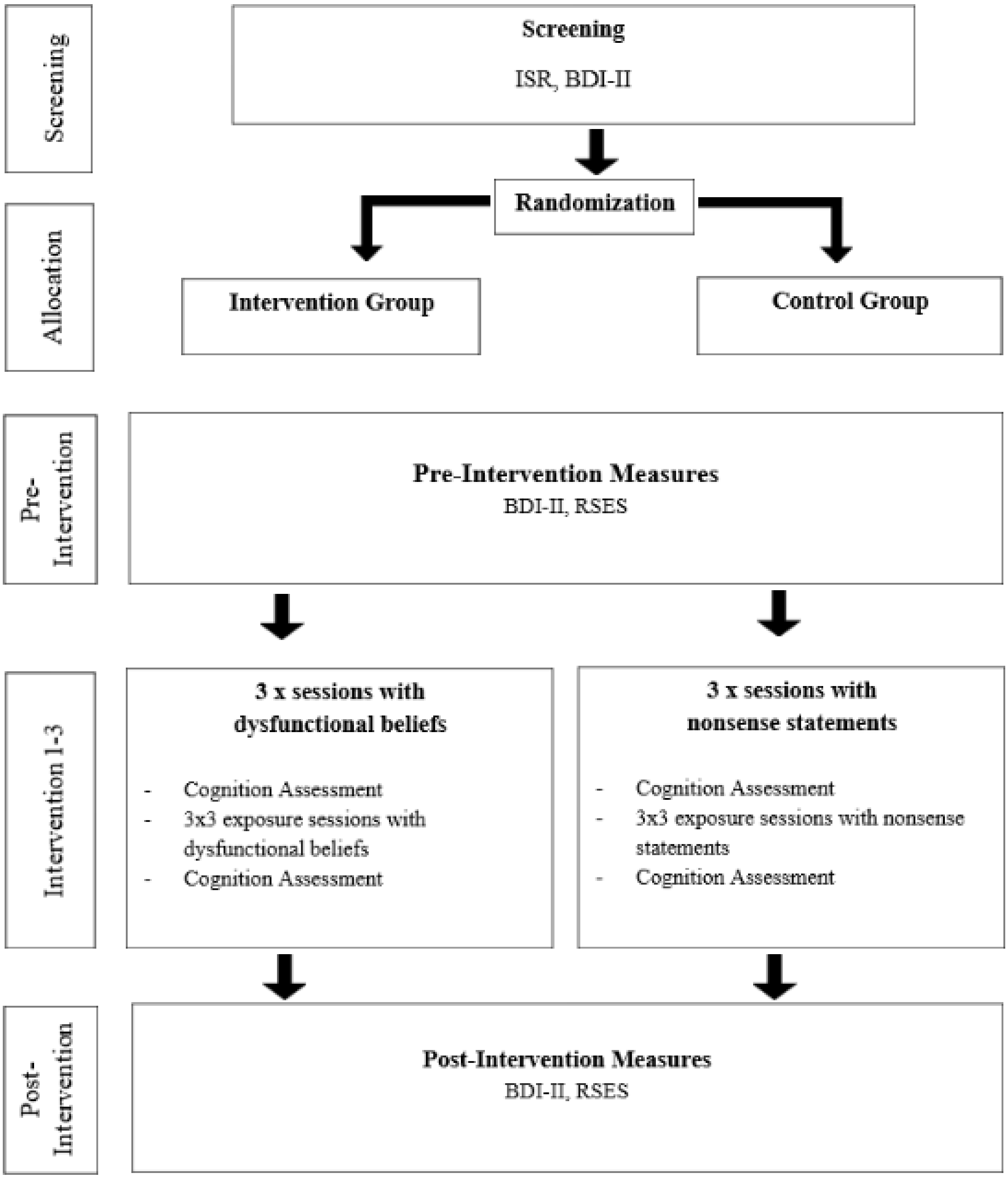

Supplementary Figure S1. Flow chart of the study's experimental procedure. *Note. ISR = ICD-10 Symptom-Rating, BDI-II = Beck-Depression-Inventory II, RSES = Rosenberg Self Esteem Scale.*

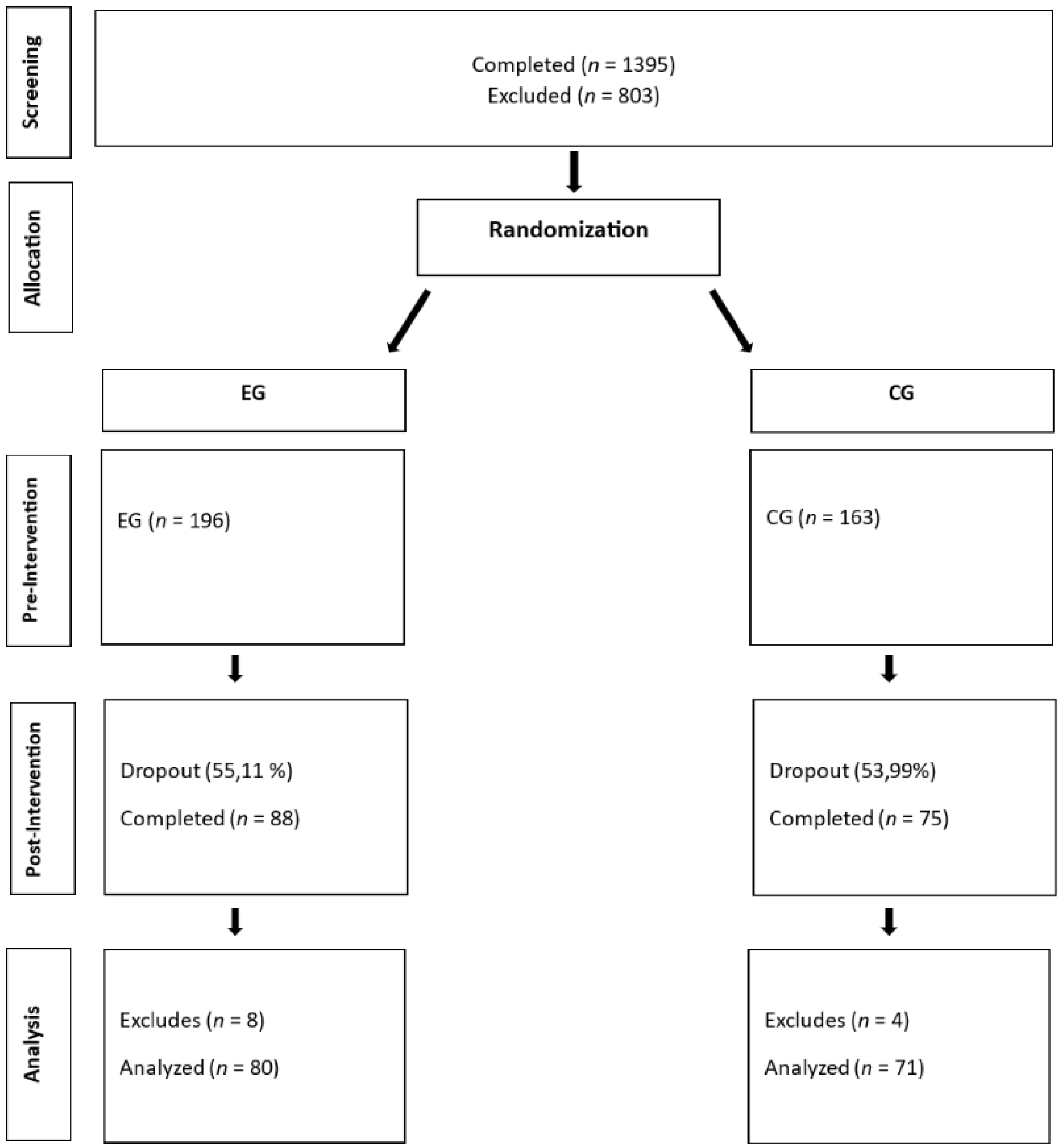

Supplementary Figure S2. Flow chart of the study's experimental procedure including the dropout.
